# Drivers of daily movement patterns affecting an endangered vulture flight activity

**DOI:** 10.1186/s12898-018-0195-7

**Published:** 2018-09-29

**Authors:** Ruth García-Jiménez, Juan M. Pérez-García, Antoni Margalida

**Affiliations:** 1 Department of Animal Science, Faculty of Life Sciences and Engineering, University of Lleida, 25198 Lleida, Spain; 2 Division of Conservation Biology, Institute of Ecology and Evolution, University of Bern, 3012 Bern, Switzerland; 3 Institute for Game and Wildlife Research (CSIC-UCLM-JCCM), Ronda de Toledo s/n, 13071 Ciudad Real, Spain

**Keywords:** Daily movements, Daylight time, GPS, *Gypaetus barbatus*, Season, Spain, Territorial status

## Abstract

**Background:**

The development of satellite tracking technology enables the gathering of huge amounts of accurate data on animal movements over measured time intervals, to reveal essential information about species’ patterns of spatial use. This information is especially important in optimizing the design of conservation and management strategies for endangered species. In this study, we analysed the main drivers of daily patterns in the flight activity of the threatened Bearded Vulture *Gypaetus barbatus*. We studied 19 Bearded Vultures tagged with solar-powered GPS transmitters from 2006 to 2016 in the Pyrenees (Spain). We assessed the relative influence of external factors (season and daylight time) and internal factors (sex, breeding season and territorial status) on their daily activity behaviour by computing mean hourly distance travelled, maximum displacement and cumulative distance travelled per hour.

**Results:**

Our findings showed a clear difference in all the estimators between territorial and non-territorial (floating) members of the population, showing that non-territorial individuals spent much longer in flight and travelled larger distances per day. We detected an important influence of daylight time and season on the daily rhythms of Bearded Vultures; flight activity increased during the last three quarters of daylight and was greatest in the spring. Breeding period and sex had also an effect on the maximum displacement and cumulative distance travelled. Individuals flew more during the breeding period and females tended to exhibit greater cumulative and maximum distances per hour than males regardless of breeding season.

**Conclusions:**

Pyrenean Bearded Vultures flight daily activity was strongly influenced by daylight time, season, and territorial status, while individual sex and breeding season showed a milder effect on the birds’ movement behaviour. This study gives a novel insight into how external factors act as main drivers of the daily flight activity pattern of a long-lived avian scavenger.

**Electronic supplementary material:**

The online version of this article (10.1186/s12898-018-0195-7) contains supplementary material, which is available to authorized users.

## Background

Interest in movement ecology has increased in recent years due to its key role in the design of more specific and efficient management and conservation strategies. The development of satellite tracking technology enables the gathering of huge amounts of accurate data on animal movement over measured time intervals, to provide essential information on species’ patterns of spatial use [1, 2]. Modern satellite transmitters can also record individual physiological parameters during flight [3, 4]. The activity decisions made by individuals influence overall population behaviour and so affect population viability as each individual decides its own specific demographic process, such as migration, feeding, and reproductive behaviour [5]. The assessment of space use and territory occupancy patterns is particularly useful in bird community studies (e.g. [6–8]). Beyond the direct information gathered on dispersal [9, 10], roost site selection [11], and foraging activity [12, 13], study of movement ecology provides information indirectly related to an animal’s behaviour in reaction to prevailing climatic conditions [4, 14, 15], on the effects of food availability on the use of space and on population trends [16, 17].

Avian scavengers provide human society with indispensable ecological services, recycling carrion biomass through their removal of waste and preventing the accumulation of dead animal biomass, so reducing the spread of diseases and contributing to nutrient cycling [18, 19]. Due to the ephemeral occurrence and random distribution of carcasses, vultures have evolved adaptive traits to exploit carrion as effectively as possible [20–22]. The balance between their maximization of food intake and minimization of energy expenditure has therefore developed to determine the daily foraging movements of scavenging species [23, 24]. To date, several internal and external factors have been suggested as drivers of the daily activity movement patterns of scavengers, acting either independently or in synergy. Intrinsic factors include biological and physiological parameters such as territorial status, sex, breeding season and level of hunger [23, 25–27]. External factors are mainly characterized by weather conditions and—both of which generally change seasonally—[4, 28], food availability [20], and intra- or interspecific interactions [20].

The Bearded Vulture *Gypaetus barbatus* is a long-lived territorial vulture inhabiting Old World mountain biomes with a diet consisting 70–90% of bones from wild and domestic ungulates, and therefore occupies a very specialized trophic niche [22, 29, 30]. Despite the increment of the Pyrenean population in the last 30 years, this positive tendency could be menaced by mortality factors such as the illegal use of poison baits, lead intoxication, food shortages, and anthropogenic habitat changes [31–35]. This situation highlights the need for an in-depth understanding of the potential threats, including mortality hotspots, the causes of breeding failure, and limitations on the species’ use of space. For instance, information regarding their daily activity patterns is especially useful in planning reintroduction conservation programs and to enhance any future conservation or management action considering its habitat use and spatial behaviour.

This study set out to assess the influence of internal and external factors on the daily activity patterns of Pyrenean Bearded Vulture flight activity. To this end, we analysed 38,248 data obtained from a population of 19 GPS-tracked Bearded Vultures in the Pyrenees (Spain) between 2006 and 2016, to examine the effect of internal factors such as sex, territorial status and breeding season, and of external factors such as daylight time and season.

## Methods

### Study species

The habitat distribution of Bearded Vultures has been shrinking since the 1970s (with only 243 pairs remaining in the European Union in 2016). During the last 30 years a variety of management and conservation programs have been developed for this threatened species, achieving a substantial rise in the Pyrenean population, although, the overall distribution of Bearded Vulture has scarcely expanded [36]. This species is enlisted as near threatened by the IUCN Red List [37].

### Study area

This study was conducted in Pyrenees, located in the border area between France and Spain, in the Eurosiberian region. In this area the Bearded Vulture population comprises more than 70% of the European breeding population. The most important breeding areas lies on the southern slopes of the Pyrenees, with the highest nesting densities in steeply sloping areas over 1000 m height level, where human access is limited and orographic updraughts are more frequent [30].

### Capture, tracking and data collection

Twenty Bearded Vultures were captured in the period 2006–2016 using radio-controlled bow-nets at supplementary feeding stations (n = 17), at nests (n = 1), or as injured individuals recovered at official wildlife recovery centres (n = 2), where birds are released following rehabilitation (for more details about these individuals’ capture see [17, 38]). We monitored their movement patterns using 70 g solar-powered Argos satellite transmitters (PTT/GPS Microwave Telemetry, Inc. Columbia, MD, USA) attached by means of a breakaway harness with a 0.64 cm Teflon ribbon (Bally Ribbon Mills, Bally, PA, USA). The transmitters were programmed to send a fix (manufacturer’s estimated error ± 18 metres) each hour from 4:00 to 22:00 UTC, with the exception of two individuals, whose transmitters sent a GPS location every 2 h. Birds were aged into four different classes using plumage characteristics: juveniles (birds until the 1st year), immatures (2–3 years), sub-adults (4–5 years) and adults (6 years or over). Identification of gender was performed using blood samples by PCR amplification of the CHD-W gene [39]. We defined territorial Bearded Vultures when exhibited spatially aggressive defense, nest-building behaviour and sexual activity on a fixed area [38–41].

### Data processing and statistical analysis

We analysed the daytime routine of Bearded Vultures by calculating three different estimators: maximum displacement, defined as the average Euclidean distance between the initial daily location and any position reached on the consecutive hours; hourly distance, approximated as the average straight-line distances covered in an hour and cumulative distance travelled, estimated as the sum of straight-line distances covered during each hour on a given day. To build a uniform and robust data base, we selected only data from days where at least seven consecutive GPS-locations were recorded during day with a maximum time lapse of 4 h between fixes. One of the tracked birds did not meet this minimum set of criteria for locations, so we exclude all its data from the analysis.

We studied differences in the daily movement parameters according to three internal factors: sex, breeding season, and territorial status; and two external factors: daylight time and season.

To evaluate the influence of sex on the daily movement of Pyrenean Bearded Vultures we considered only territorial individuals. For breeding season comparisons of daily activity patterns, we divided the data in the two breeding periods (*breeding period*, from 1st January to 31st July, and the *non*-*breeding period*, from 1st August to 31st December) based on Margalida et al. [38]. To study the possible influence of season on the daily pattern of flight activity we defined four seasons conforming to the Mediterranean climate: spring (from 21st March to 20th June); summer (from 21st June to 22nd September); fall (from 23rd September to 20th December); and winter (from 21st December to 20th March). We did not include age in the analysis because our previous studies showed it to be subordinate compared to territorial status [38]. Differences in maximum displacement, cumulative distance travelled and hourly distance travelled for different territorial status and breeding season were compared using the Wilcoxon Mann–Whitney tests. Sex related differences between territorial individuals were also tested for these three variables. We analysed each relationship independently.

To standardize the seasonal variation in daylight, we generated an index of daylight duration (hereinafter called daylight index) which denotes the daylight time considering the astronomical twilight as the start and the end of a daylight length setting sunrise -the astronomical dawn, the time when the geometric centre of the Sun is 18 degrees below the horizon in the morning—(value 0) and sunset—the astronomical dusk, when the geometric centre of the Sun is 18 degrees below the horizon preceding the night—(value 1) for each day. We included the three twilight periods before sunrise (astronomical, nautical and civil twilights; data obtained from www.timeanddate.com and summarised in Additional file 1) because several authors have suggested that they mark the beginning of the first daily peak of activity in bird’s circadian pattern [42–44], as well as a short time after sunset during which birds were observed making the journey back to their roosting sites. We computed this daylight index as the division of daylight elapsed fix time by daylight length, where the numerator is the period of daylight spent until the fix transmission, and denominator is length of daylight hours within a given 24 h day.

To analyse and represent the data we grouped the daylight index ranges into an integer scale from 0 to 10 following the scale described above, but to a higher decimal order. We incorporated also some locations before and after the astronomical twilight (with index values − 1 and 11, consecutively) to evaluate the behaviour of the birds some dark hours previous to sunlight incidence (Additional file 1, Additional file 2: Figure S1 and Table S1).

To examine the relationship between movement parameters and biological (sex, breeding season, and territorial status) and external (daylight time and season) factors we used linear mixed models (LMM) with individual as a random factor [45]. We compared each model with the null case, including both the variables and the interactions. Model comparisons were carried out using Akaike information criteria (AICc; [46]). We computed delta AICc to determine the strength of evidence, and AICc weights to represent the relative likelihood of each model [46]. Models with delta AICc > 4 were discarded. All analyses were conducted using R statistical software (v 2.3-2. R Development Core Team 2007, http://www.R-project.org) with the lme4 package for LMM analyses. All tests were two-tailed and statistical significance was set at α ≤ 0.05. All results were shown as mean ± 1 SD.

## Results

We recorded 78,814 GPS locations from 20 Pyrenean Bearded Vultures, during November 2006 to December 2016. After filtering, we analysed 38,248 fixes from 19 individuals. The highest frequencies of locations were recorded from 9:00 to 16:00 UTC usually concurring with the hours with major sunlight availability (Additional file 2: Figure S1, S2 and Table S2). The records were—according to sex—34.1% females and 65.9% males and—in terms of the age class and territorial status—86.6% adults (of which 28.6% were locations from territorial birds), 11.3% were from subadults, 2.0% were from immatures, and 0.1% were from juveniles.

### Territorial status and breeding season

**Fig. 1:**
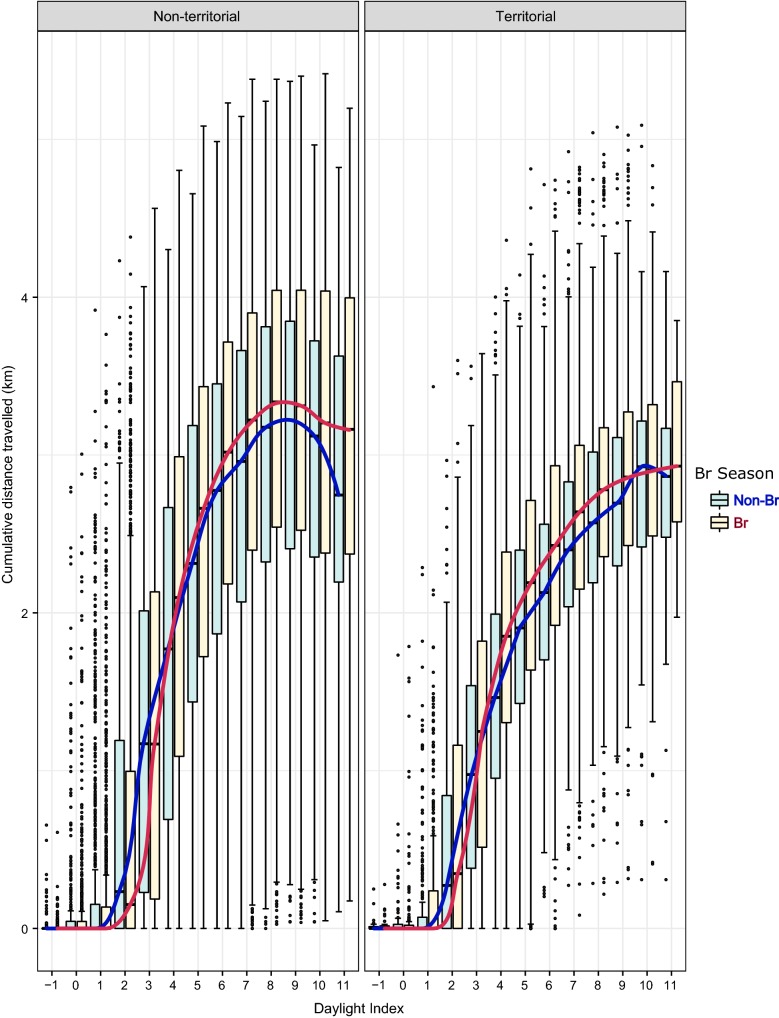
Influence of territorial status (left: non-territorial, right: territorial) and breeding season (blue: non-breeding, red: breeding) on the cumulative distance travelled. The response variable, log (y + 1), has been transformed to represent the variation graphically

**Fig. 2:**
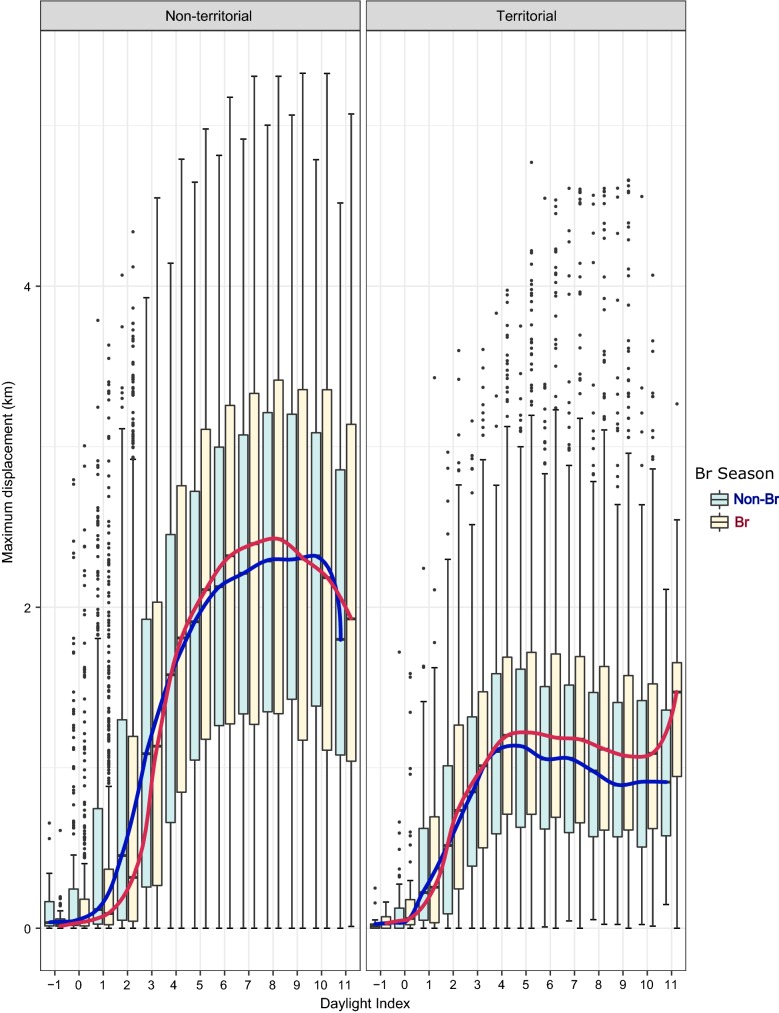
Influence of territorial status (left: non-territorial, right: territorial) and breeding period (blue: non-breeding, red: breeding) on the maximum displacement travelled by adult territorial individuals

**Fig. 3:**
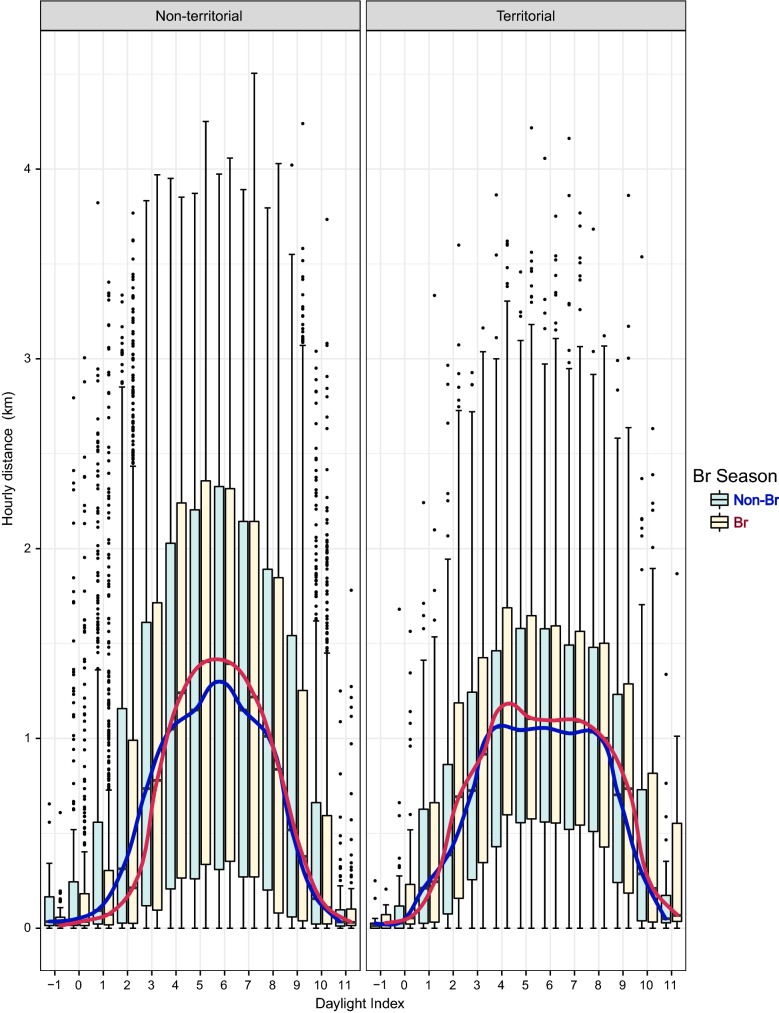
Influence of territorial status (left: non-territorial, right: territorial) and breeding period (blue: non-breeding, red: breeding) on the hourly maximum displacement. The response variable, log (y + 1), had been transformed to represent the variation graphically

The floating population (non-territorial birds) exhibited a significantly greater daily activity pattern compared to territorial birds. Significant differences were found in cumulative distance travelled (Wilcoxon test, Z = 13.0, p < 0.001), maximum displacement during the daylight (Z = 40.2, p < 0.001) and hourly distance travelled (Z = − 3.4, p < 0.001) according to their territorial status. Non-territorial individuals exhibited the highest values for the three daily distance covered estimators during the breeding period (Figs. 1, 2 and 3). In non-territorial individuals, the maximum mean cumulative distance travelled was c. 42 km, showing a marked rise during the two middle daylight quarters (from daylight index values of 2–8), while territorial individuals showed a gradual increase in this distance estimator throughout the daylight hours (Fig. 1), reaching maximum medium values of 20–22 km cumulative distance travelled. The same pattern was observed for the maximum daytime displacement in the non-territorial birds, although territorial vultures showed increasing mean values until the middle of the daylight period, followed by stabilization of these values (Fig. 2). Independently of territorial status, the longest average hourly distances were travelled during the middle of the daylight period, although the greatest distances were achieved by non-territorial individuals (6.75 ± 9.05 km), regardless of breeding season (Fig. 3). Furthermore, non-territorial individuals during the breeding period showed a range of maximum average displacements between 0.06 ± 0.11 and 20.77 ± 26.51 km, while non-breeding birds had a significantly lower mean maximum displacement range of between 0.14 ± 0.21 and 16.83 ± 21.01 km (Z = − 7.4, p = 0.01). Breeding season also significantly affected territorial individuals: during the breeding period they exhibited a notably higher maximum distance from the nest 5.25 ± 13.56 km, and longer mean cumulative distance travelled of 22.07 ± 21.48 km, compared to the maximum daily displacement of 3.72 ± 8.41 km (Z = − 7.5, p < 0.001) and daily covered distance of 20.02 ± 18.06 km (Z = − 5.7, p < 0.001) observed during the non-breeding period. The territorial birds also showed significantly higher values of hourly displacement during the breeding period (Z = − 4.6, p < 0.001; see Fig. 3).

### The effect of sex in territorial Bearded Vultures

**Fig. 4:**
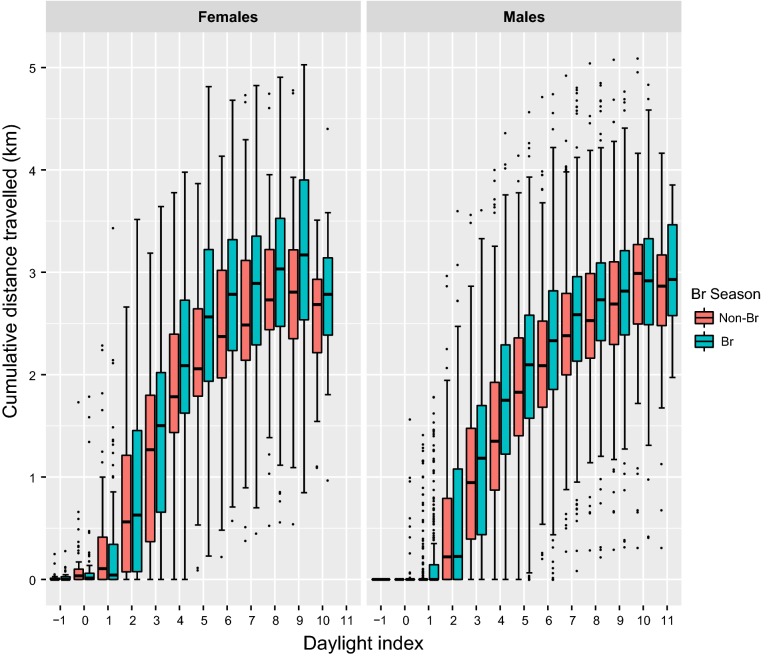
Influence of sex and breeding period (red: non-breeding, blue: breeding) on the cumulative distance travelled by adult territorial individuals

**Fig. 5:**
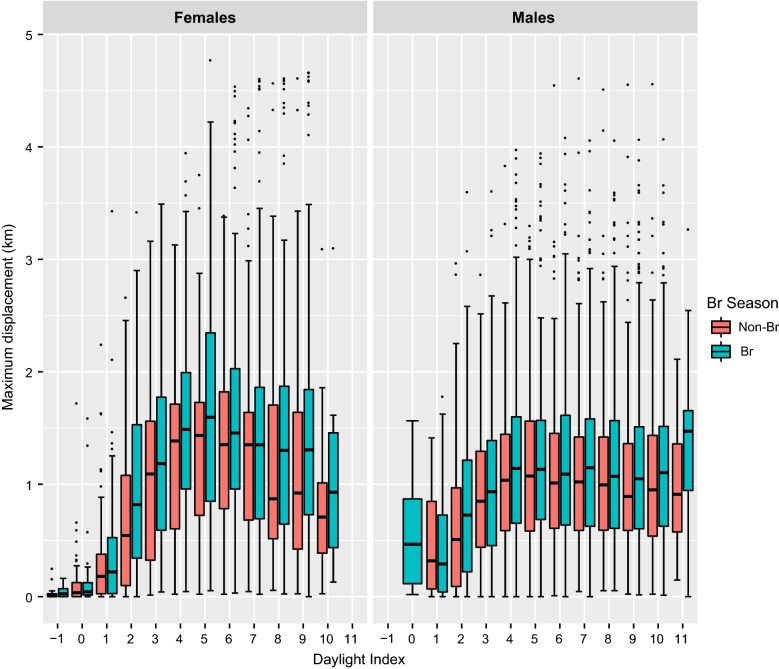
Influence of sex and breeding period (red: non-breeding, blue: breeding) on the maximum displacement travelled by adult territorial individuals

**Fig. 6:**
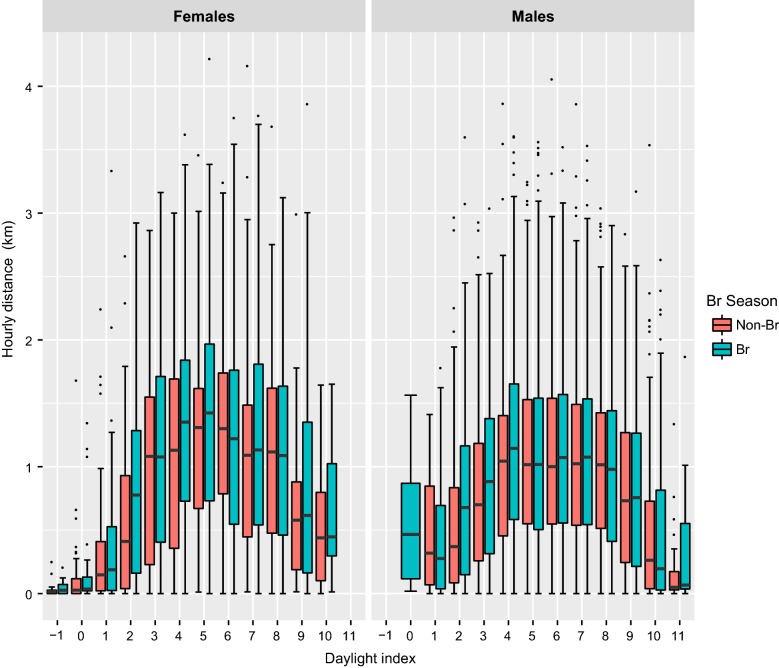
Influence of sex and breeding period (red: non-breeding, blue: breeding) on the hourly mean distance travelled by adult territorial individuals

During the breeding period, females showed higher flight activity than males, performing maximum distances travelled per day of 14.31 ± 28.93 km, cumulative distances travelled of 37.38 ± 37.45 km, and hourly distances travelled of 5.22 ± 7.45 km, in contrast with males which travelled mean maximum day distances of 5.07 ± 6.76 km (Z = 5.2, p < 0.001), cumulative daily distances of 21.67 ± 17.53 km (Z = 2.4, p = 0.02) and hourly distances of 3.24 ± 4.27 km (Z = 3.3, p = 0.001). A similar trend was also observed within the non-breeding birds, where males achieved a maximum displacement of 3.20 ± 4.12 km and hourly distances of 3.04 ± 3.94 km at least 1 km significantly less than females, which achieved maximum distances covered per day of 6.96 ± 17.94 km (Z = − 2.6, p = 0.009) and hourly movements of 4.42 ± 5.48 km (Z = − 3.0, p = 0.002; see Figs. 4, 5 and 6).

### Seasonal patterns

Based on the linear mixed models results, season is a highly significant factor leading to remarkable differences between the mean seasonal values of all three flight distance estimators (Table 1).

**Table 1:** Linear mixed models to explore the factors influencing the distance covered estimators (maximum displacement, cumulative distance travelled and hourly distance)

Model	Factors	K	AICc	ΔAICc	W
Maximum displacement	Territ* *Sex*+*Season*+*DI*	10	101,320.8	0.00	0.99
Cumulative travelled distance	*Territ*+*Season*+*DI*+*Sex*+*Br*_*S*	10	107,447.1	0.00	0.65
	Territ + Season + DI	8	107,449.3	2.28	0.21
Hourly distance	*Territ*+*Season*+*DI*	8	89,516.3	0.00	0.64
	Territ + Season + DI + Sex	9	89,518.2	1.93	0.24
	Territ + Season + DI + Sex + Br_S	10	89,519.6	3.29	0.12

Factors included were territorial status (*Territ*), daylight index (*DI*), climatic season (*Season*), breeding season (*Br_S*), and sex (*Sex*) and the simple interactions Sex*Territ, Season*Territ and Br_S*Territ. The model with the lowest AIC value (in italics) is the most parsimonious. K: total number of parameters (explanatory terms + random term + residual deviance); AICc: corrected Akaike information criterion; ΔAICc: difference between the AICc value for that model and the best model; and, W: Akaike weights

**Fig. 7:**
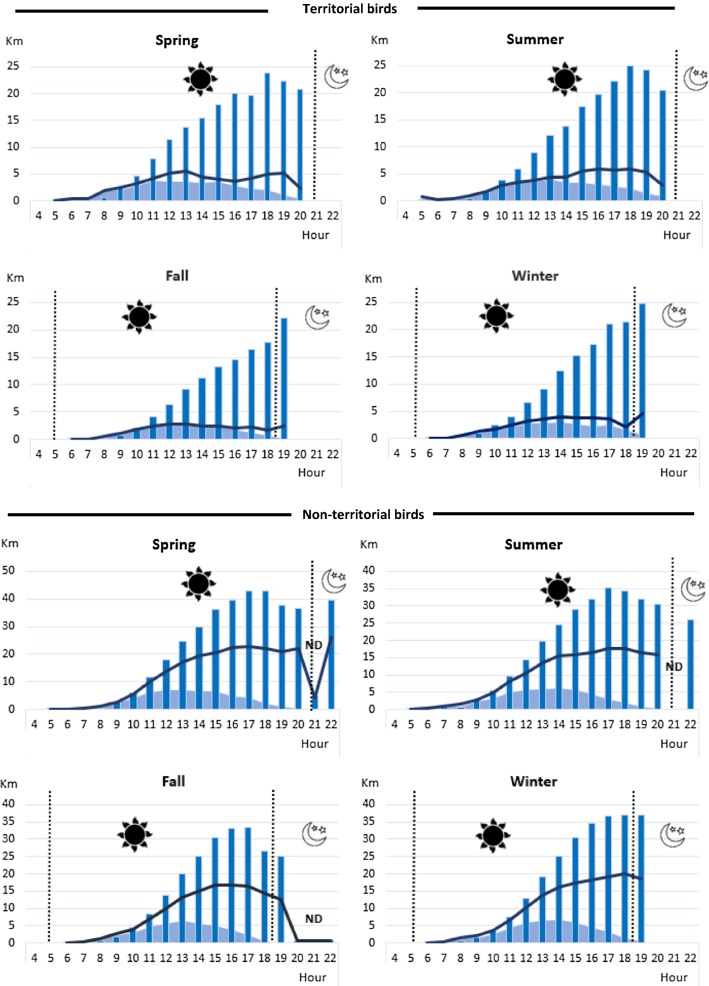
Influence of season and territorial status on Bearded Vultures flight activity represented by three estimators: maximum displacement (line chart), cumulative distance travelled (bar chart) and hourly distance travelled (area chart). *ND* not enough data available at that level. Astronomical twilight is marked—if it is present—with a dashed line. The sun is placed at noon time

Flight activity of non-territorial birds stands out in spring, when they reached the greatest maximum daytime displacement, cumulative distance travelled, and hourly distance. Nevertheless, a similar flight pattern was observed for non-territorial Bearded Vultures in every season, showing a growing trend for the daily maximum displacement and cumulative distance travelled from 8 h since 18 h (UTC), excepting fall, when the peak of activity was achieved a little before (around 16–17 h, UTC). In spring and summer (the two seasons with the highest daylight availability) the Bearded Vulture flight activity extended longer (until 22 h UTC). The second greatest maximum displacement and cumulative distance travelled was recorded in winter (Fig. 7).

Territorial Bearded Vultures presented an increased flight activity during spring and summer achieving the peak approximately at 18 h UTC. In fall and winter even though the flying activity decreased, the rise was interestingly detected at 19 h, coinciding with the hours around astronomical sunset. No data were registered after 20 h for territorial birds (Fig. 7).

Concerning hourly distance, all the individuals showed a uniform movement pattern during all the year, attaining the maximum values around 13 h UTC. During fall, individuals travelled the shortest distances (Fig. 7).

### Multifactorial model

The daylight index and seasonal factors were the most influential of all the parameters tested in every linear model since they were selected in each of the models built for the three distance covered estimators. Consecutively, territorial status had the next most noticeable effect on cumulative distance travelled and hourly displacement, followed by the effect of sex which only appeared in the cumulative distance travelled model. Breeding season was the factor with the weakest relationship with all of the three distance covered estimators.

The best explanatory model for maximum displacement involved the interaction between sex and territorial status, daylight index, and seasonal variables. In the hourly distance case, the model comprising territorial status, season and daylight index overcame the null model, while for the cumulative distance travelled estimator, the best model involved all of the variables tested (Table 1, Additional file 3).

## Discussion

Our results on daily flight behaviour show an important spatial decoupling between the territorial and non-territorial individuals in the Pyrenees. Because non-territorial individuals are not central place foragers, they exhibited greater daily flight activity travelling longer distances, showing greater cumulative distances covered in an hour, higher maximum displacements, and greater hourly distance rate. These findings agree with the results regarding foraging movements obtained by Krüger et al. [26] in South Africa and by Margalida et al. [38] in the Pyrenees, in which territorial status influenced spatial distribution patterns of Bearded Vultures. In these studies, non-territorial individuals exhibited Kernel 90% home ranges of between 10,500–26,000 km^2^ in South Africa and 1800–11,600 km^2^ in the Pyrenees, areas that are significantly larger than those covered by territorial individuals of 286 ± 361 km^2^ in South Africa and 63 ± 59.5 km^2^ in the Pyrenees. In addition, our results show a daily temporal dissociation according to the status of an individual (territorial vs non-territorial); non-territorial birds showed greater increments in maximum distance covered and the cumulative distance covered. The non-territorial status of these individuals allows them to travel farther and until later into the daylight period (i.e. during the last third of the daylight hours) compared with territorial individuals, who increased their maximum daily distance travelled until the period close to noon after which their daily maximum distance values stabilised (Figs. 1, 2, 3, 4, 5, 6).

Our findings suggest that breeding period also has an influence over the daily flight activity, but lower than other internal factors. As with other obligate avian scavenger species, breeding Bearded Vultures experience an increased energy requirement due to parental effort. These reproductive tasks could explain the noticeable rise in the three different distance parameters measured during the last three quarters of the daylight period in the territorial birds. This accords with the significant seasonal effect detected in their daily activity patterns because the greatest distance of maximum displacement, cumulative distance and hourly distance travelled were observed in spring -especially for non-territorial birds -, coinciding with the peak of the breeding period, whilst the shortest were realized in fall during the non-breeding period (Fig. 7). However, our results only showed a significant effect of the breeding period on the cumulative distance travelled. Reproductive failure is a factor which should also be considered because it would allow the vultures to travel further afield, especially during March and April, when reproductive failure rates (hatching period and first days of the chick) are at their highest.

The influence of season has been generally evident in other studies of the circadian rhythm of birds [24, 47, 48], because variations in the quantity and intensity of solar radiation throughout the year determinate the timing of a bird’s circadian behaviour [49], and conditioning intrinsic factors such as the speed of migration [50]. Seasonal effects can also influence external factors such as variation in carrion food availability due to seasonal transhumance of livestock [17], thus shaping vultures’ daily activity patterns, and biasing the performance of solar powered GPS transmitters [51]. We detected a seasonal influence on the values of the distance covered estimators, the longest distances being recorded in spring. Flight activity pattern seems to increase similarly on every season during the same daylight time (at the last third of the daylight) differentiating between the two territorial status. An elevated flight activity is maintained by non-territorial individuals some hours after astronomical sunset for every season, whilst territorial birds seem to sustain or even augment their activity pattern after dusk particularly in winter (but not in spring or summer), probably related with the reproductive period (Fig. 7). In addition, the flying fixes ratio registered in winter (37%) was higher than all of the other seasons (the lowest was logged in summer (32.5%), considering flying fixes > 1.39 m/s following Silva et al. [51]). This supports our aforementioned hypothesis that the energetic requirements of Bearded Vultures rise during the breeding period (winter and spring) combined with the decrease in of food availability in this time of year [17] which forces them to fly for longer periods and over longer distances. However, despite this, we did not observe a clear seasonal variation in the daily activity patterns of the territorial Bearded Vulture flight behaviour.

While timing of sunrise and sunset determines the daily start and end of aerial activity in most obligate scavengers [52], Bearded Vulture is able to continue flying after the sunset. In fact, maximum air temperature and wind speed in temperate climate ecosystems, and thus the best wind uplift conditions for large avian scavenger flight, occur in summer during the hours around noon [49, 53]. So, even while the greatest chances of finding profitable carcasses are in the early morning hours because ungulate mortality peaks during the night [20, 24], the highest displacements of Pyrenean Bearded Vultures are recorded during the second half of the daylight, regardless of season, by virtue of their energy-efficient foraging flight and reduced wing loading in comparison with other vulture species [15, 49, 54]. This allows Bearded Vultures to profit the later daylight hours of convective updraughts to return to the nest or to search for a roosting site [15]. Moreover, the specific diet of this vulture—based mainly on the exploitation of bone remains, a resource which is preserved long time after a carcass has died—[29, 55] releases it from interspecific competitive pressures, reasonably diminishing the impact of the optimal time to exploit carrion in the species daily feeding habits [22]. All these physiognomical and ecological attributes enable Bearded Vultures to solve the trade-off between the ideal feeding time and the availability of wind resource performing the furthest travelling distances during the afternoon, even though the greatest hourly distances travelled are achieved at mid-day.

The sex of an individual influenced the longest distances covered in a day and our results showed intra-sexual, but not inter-sexual, differences for this estimator. Concretely, both adult non-territorial females and males travelled significantly farther in a day than territorial individuals. However, an unexpected asymmetry was detected between the sexes for the cumulative distance travelled. Females covered significantly more kilometres than males during a day, consistent with the trend in spatial use already described for the same Pyrenean Bearded Vulture population [38]. Several studies of avian species underpin this inter-sexual spatial pattern discordance relating to the behavioural differences in reproductive roles between the sexes [56, 57] as well as individual or even sex-size variations [24, 58, 59]. However, the Bearded Vulture is a monomorphic species and parental care is divided equally between the male and female [60], and therefore we would predict similar energy requirements for both sexes. A possible explanation of this sexual difference in daily distance covered during the breeding period could be due to the raised female energy demand resulting from the egg biosynthesis and the reproductive jeopardy if this is not met. In spite of the differences in daily spatial behaviour between the sexes, there are no differences in the temporal daily flight patterns between them.

According to our findings, the daytime flight behaviour of the Bearded Vulture does not follow a random pattern. The external factors studied (daylight index and season) strongly regulate the daily flight activity, while internal factors such territorial status, sex, and breeding period mould its flight dynamic. The synergy between both categories of factors enables the Bearded Vulture to confront the trade-off between travel costs—mostly constrained by weather conditions—and energy requirements. In addition, territorial status was, predictably, the most influential of all the internal factors studied. Other interesting drivers of flight behaviour have come to light, such as the relationship between territoriality and breeding season and the influence of sex in this monomorphic species, suggesting that these synergistic and intrinsic factors may play a currently unexplored role in this species’ flight patterns.

Understanding the daily movement ecology of the Bearded Vulture is essential for predicting its future dispersal, foraging and reproductive patterns. These data are interesting for developing future conservation strategies (such as those related to the management of Supplementary Feeding Sites) both in the Pyrenean region and other ecosystems with distinct climatological conditions or food availability. Indeed, given the variety of mortality risks faced by this species and its high adult mortality rate [32, 36, 61], information on the daily distances travelled by juveniles during their early dispersal stages might help to improve the design of future conservation measures.

## Conclusions

This study is the first detailed daily activity analysis developed on the Bearded Vulture improving our knowledge on the movement ecology of this threatened species trough a finer spatio-temporal information about the daytime flight routine of the species. Our findings show that the main drivers of the Bearded Vulture daily flight activity are daylight time, season, and territorial status of the individual. This agrees with several authors’ hypothesis supporting the daylight time as the most influential factor of all of the external factors determining circadian behaviours [62]. Pyrenean Bearded Vultures covered the furthest travelling distances during the afternoon. Moreover, internal factors as territorial status had a remarkable effect on the daily activity patterns of the vulture. Non-territorial Bearded Vultures presented the greatest daily flight patterns. Both individual’s sex and breeding period mildly shaped the flight activity resulting in the females and breeding individuals travelling further afield than males and non-breeding individuals.

## Additional files

**Additional file 1:** Mean ± SD values for the three twilight phases, the solar noon and the daylength for each season in UTC Time (hh:mm). Source: www.timeanddate.com.

**Additional file 2: Figure S1.** Frequencies of fix per daylight percentage range. **Table S1.** Table of frequencies of fix per daylight percentage range. **Figure S2.** Frequencies of fix per season considering UTC Time.

**Additional file 3:** Standardized weights of all the predictors introduced in the linear mixed models performed (N models).
