# Supplementary material for: Drivers of daily movement patterns affecting an endangered vulture flight activity
Source: BMC Ecol. 2018 Sep 29;18:39. doi: 10.1186/s12898-018-0195-7 (PMC6162909; doi:10.1186/s12898-018-0195-7)
Supplement: Supplementary file 1 — Additional file 1: Mean ± SD values for the three twilight phases, the solar noon and the daylength for each season in UTC Time (hh:mm). Source: www.timeanddate.com. [file 12898_2018_195_MOESM1_ESM.docx]

**Additional file 1:** Mean ± SD values for the three twilight phases, the solar noon and the daylength for each season in UTC Time (hh:mm). Source: www.timeanddate.com .

|  | **Astronomical twilight** | | | | | | |  |
| --- | --- | --- | --- | --- | --- | --- | --- | --- |
|  |  | **Nautical twilight** | | | | |  |  |
|  |  |  | **Civil twilight** | | |  |  |  |
|  | Sunrise | Sunrise | Sunrise | Noon | Sunset | Sunset | Sunset | **Daylenght** |
| **Spring** | 2:56 ± 0:45 | 3:40 ± 0:38 | 4:19 ± 0:34 | 14:05 ± 0:03 | 19:27 ± 0:29 | 20:06 ± 0:34 | 20:50 ± 0:41 | 13:48 |
| **Summer** | 2:58 ± 0:40 | 3:42 ± 0:33 | 4:21 ± 0:29 | 14:05 ± 0:04 | 19:29 ± 0:35 | 20:08 ± 0:39 | 20:52 ± 0:46 | 14:08 |
| **Fall** | 4:55 ± 0:27 | 5:29 ± 0:28 | 6:02 ± 0:29 | 10:16 ± 0:04 | 17:19 ± 0:27 | 17:52 ± 0:26 | 18:26 ± 0:25 | 10:36 |
| **Winter** | 5:18 ± 0:24 | 5:51 ± 0:25 | 6:25 ± 0:26 | 10:14 ± 0:05 | 17:39 ± 0:32 | 18:13 ± 0:31 | 18:46 ± 0:30 | 10:16 |
