# Supplementary material for: Drivers of daily movement patterns affecting an endangered vulture flight activity
Source: BMC Ecol. 2018 Sep 29;18:39. doi: 10.1186/s12898-018-0195-7 (PMC6162909; doi:10.1186/s12898-018-0195-7)
Supplement: Supplementary file 2 — Additional file 2: Figure S1. Frequencies of fix per daylight percentage range. Table S1. Table of frequencies of fix per daylight percentage range. Figure S2. Frequencies of fix per season considering UTC Time. [file 12898_2018_195_MOESM2_ESM.docx]

**Additional file 2**

**Additional file 2: Figure S1.** Frequencies of fix per daylight percentage range.

**Additional file 2: Table S1.** Table of frequencies of fix per daylight percentage range.

| **Integer scale** | **Daylight units** | **N fixes** |
| --- | --- | --- |
| -1 | -0.1 - 0 | 20 |
| 0 | 0 - 0.1 | 418 |
| 1 | 0.1 - 0.2 | 1795 |
| 2 | 0.2 - 0.3 | 3998 |
| 3 | 0.3 - 0.4 | 5154 |
| 4 | 0.4 - 0.5 | 4688 |
| 5 | 0.5 - 0.6 | 5811 |
| 6 | 0.6 - 0.7 | 5458 |
| 7 | 0.7 - 0.8 | 4832 |
| 8 | 0.8 - 0.9 | 4187 |
| 9 | 0.9 – 1 | 1690 |
| 10 | 1 – 1.1 | 197 |
| 11 | 1.1 - 1.2 | 20 |
| **Total** |  | **38248** |

**Additional file 2: Figure S2.** Frequencies of fix per season considering UTC Time.
