# Supplementary material for: Drivers of daily movement patterns affecting an endangered vulture flight activity
Source: BMC Ecol. 2018 Sep 29;18:39. doi: 10.1186/s12898-018-0195-7 (PMC6162909; doi:10.1186/s12898-018-0195-7)
Supplement: Supplementary file 3 — Additional file 3: Standardized weights of all the predictors introduced in the linear mixed models performed (N models). [file 12898_2018_195_MOESM3_ESM.docx]

**Additional file 3:** Standardized weights of all the predictors introduced in the linear mixed models performed (N models) to explore the factors influencing the distance covered estimators (maximum displacement, cumulative distance travelled and hourly distance). Factors included were daylight index (*DI*), climatic season (*Season*), territorial status (*Territ*), breeding season (*Br_S*), and sex (*Sex*) and the simple interactions *Sex:Territ*, *Season:Territ* and *Br_S:Territ*.

|  | **Importance** | | | **N models** |
| --- | --- | --- | --- | --- |
|  | **Max. displacement** | **Cum. travelled dist.** | **Hourly dist.** |  |
| **DI** | 1 | 1 | 1 | 10 |
| **Season** | 0,98 | 1 | 1 | 6 |
| **Territ** | 1 | 0,83 | 0,99 | 12 |
| **Sex** | 1 | 0,61 | 0,35 | 7 |
| **Br_S** | 0,02 | 0,46 | 0,1 | 6 |
| **Sex:Territ** | 1 | <0.01 | <0.01 | 4 |
| **Season:Territ** | <0.01 | <0.01 | <0.01 | 1 |
| **Br_S:Territ** | <0.01 | <0.01 | <0.01 | 1 |
